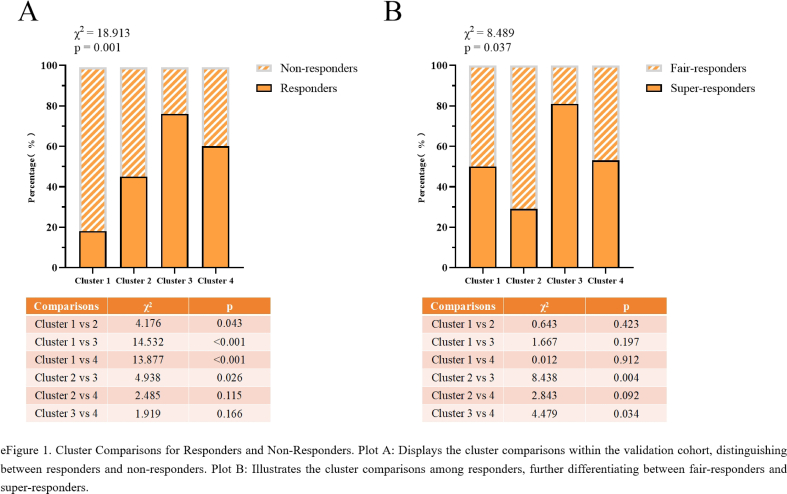# Distinct treatment response trajectories to allergen immunotherapy in allergic asthma and rhinitis: Insights from a multicenter study in routine clinical practice^☆^

**DOI:** 10.1016/j.waojou.2025.101168

**Published:** 2026-01-05

**Authors:** Pingan Zhang, Rundong Qin, Weixi Zhang, Yungang Yang, Huabin Li, Xiaoyan Dong, Yong He, Huiying Wang, Zhimin Chen, Liang Chen, Jinzhun Wu, Yanmin Bao, Man Tian, Guolin Tan, Jing Ye, Meiling Jin, Yi Liang, Kang Xu, Lijuan Mao, Qingqing Lv, Yi Zhang, Wanjun Wang, Jing Li

**Affiliations:** a State Key Laboratory of Respiratory Disease, National Clinical Research Center for Respiratory Disease, Guangzhou Institute of Respiratory Health, Department of Allergy and Clinical Immunology, The First Affiliated Hospital of Guangzhou Medical University, Guangzhou, Guangdong, China; b Department of Pediatric Allergy and Immunology, The Second Affiliated Hospital and Yuying Children's Hospital of Wenzhou Medical University, Wenzhou, 325027, China; c Department of Pediatrics, The First Affiliated Hospital of Xiamen University, School of Medicine, Xiamen University, Xiamen, Fujian, China; d Allergy Center, Department of Otolaryngology, Affiliated Eye and ENTHospital, Fudan University, No. 83, Fenyang Road, Shanghai 200031, China; e Department of Pulmonology, Shanghai Children's Hospital, School of Medicine, Shanghai Jiao Tong University, Shanghai 200062, China; f Department of Otorhinolaryngology, The First Affiliated Hospital of Ningbo University, 59 Liuting Street, Ningbo, Zhejiang, 315000, China; g Department of Allergy and Clinical Immunology, The Second Affiliated Hospitao of Zhejiang University School of Medicine, Zhejaing province, 310009, China; h Department of Pulmonary Medicine, Children's Hospital, Zhejiang University School of Medicine, National Clinical Research Center for Child Health, Hangzhou 310052, China; i Department of Allergy, Huazhong University of Science and Technology Union Shenzhen Hospital, China; j Department of Pediatric, Women and Children's Hospital, School of Medicine, Xiamen University, China; k Department of Respiratory Medicine, Shenzhen Children's Hospital, Shenzhen, Guangdong, China; l Children's Hospital of Nanjing Medical University, China; m Department of Otolarryngology, Third Xiangya Hospital of Central South University, Changsha, Hunan, China; n Department of Otorhinolaryngology, The First Affiliated Hospital of Nanchang University, 17 Yongwaizheng Street, Nanchang, Jiangxi, 330006, China; o Departmenet of Allergy, Zhongshan Hospital, Fudan University, Shanghai, 200032, China; p Department of Pediatrics, Xinchang County Hospital of Traditional Chinese Medicine, China; q Department of Pediatrics, Qingtian County People's Hospital, China; r Department of Pediatrics, The Fourth Affiliated Hospital Zhejiang University School of Medicine, China; s Department of Pediatric Internal Medicine, Taizhou Hospital of Zhejiang Province Affiliated to Wenzhou Medical University, Taizhou, 317000, China; t Department of Pediatric Respiratory Medicine, Guangzhou Yuexiu Children's Hospital, China

**Keywords:** Desensitization, Immunologic, Asthma, Cluster analysis, Routinely collected Health Data, Clinical study

## Abstract

**Background:**

While allergen-specific immunotherapy (AIT) is recognized as an effective treatment, its efficacy varies widely. However, whether clinical response trajectories to AIT differ among individuals and influence its effectiveness has not been investigated.

**Objective:**

This study aimed to characterize real-world clinical response trajectories to three-year AIT (3y-AIT).

**Methods:**

We conducted a retrospective multicenter study across 53 centers to identify clinical response trajectories in patients with house dust mite allergic asthma and rhinitis undergoing three-year AIT. The efficacy of AIT was primarily assessed using the Visual Analog Scale (VAS) for allergic symptoms at 4 time points: baseline (before AIT), and at 1, 2, and 3 years of treatment. Clustering analysis based on VAS changes at these time points was used to define response trajectories. Initial analysis was performed using data from 52 centers (Alliance cohort), and validation was conducted using data from a separate center (Guangzhou cohort).

**Results:**

In the Alliance cohort, 4 distinct clinical response trajectories were identified. Cluster 1 showed symptom worsening in the first year, with no improvement by year 3. Cluster 2 exhibited symptom deterioration in the second year, followed by significant recovery and a positive response by year 3. Clusters 3 and 4, characterized by higher and lower baseline symptom severity, respectively, demonstrated marked improvement after 3 years of AIT. In the Guangzhou cohort, a similar pattern of 4 response trajectories was observed: higher baseline symptom severity and family tobacco exposure were key features of Cluster 1 (p < 0.001), while Cluster 2 had the highest rate of respiratory infections (>1/year, p < 0.001). Despite these distinct trajectories, first-year effectiveness emerged as an ideal predictor of the 3-year AIT response, with an AUC of 0.75.

**Conclusion:**

This study identified 4 primary treatment response trajectories to 3-year AIT in daily clinical practice, highlighting the heterogeneous nature of AIT responses among individuals. Notably, first-year effectiveness appears to be an ideal predictor of the 3-year AIT outcome.

## Graphical abstract

Clinical response trajectories during a 3-year AIT for house dust mite allergic asthma were identified and validated through cluster analysis, uncovering four distinct patterns with unique characteristics.

**Figure:**
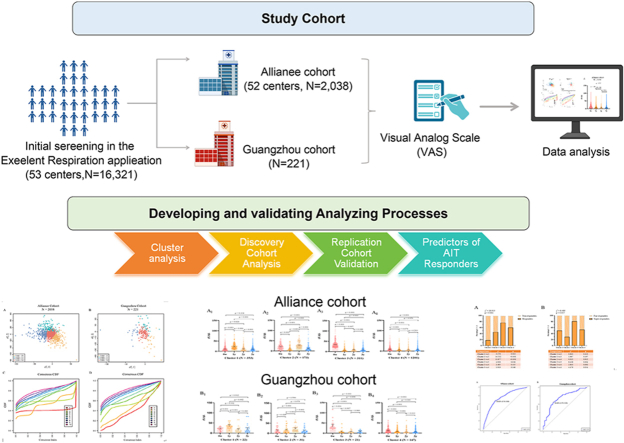

## Introduction

Allergen immunotherapy (AIT) is the only causative treatment for allergies that can modify the immune response, offering the potential to alter the natural course of allergic diseases such as allergic rhinitis and asthma.^1^, ^2^, ^3^, ^4^ Its efficacy has been well-established, with robust randomized controlled trials (RCTs) and real-world studies demonstrating significant reductions in allergic symptoms, decreased reliance on medication, and improved lung function.^5^, ^6^, ^7^

The terms “responder” and “non-responder” are commonly used to categorize patient outcomes in AIT, where responders show marked symptom relief and decreased medication use.^8^ However, pinpointing consistent predictors of AIT response remains a significant challenge. This difficulty arises from the complex interplay between allergen variability, the regulatory dynamics of both innate and adaptive immune systems, and external environmental influences.^1^^,^^4^^,^^9^ As a result, accurately predicting which patients will respond to AIT based solely on biomarkers or clinical parameters has proven difficult.^10^

Clinical response is the most direct indicator of AIT effectiveness and is shaped by factors such as the immune response to allergen extracts, treatment management, medication adherence, and environmental control. A minimum treatment duration of 3 years is recommended for AIT to establish sufficient allergen tolerance.^11^^,^^12^ Given the extended nature of AIT, regular monitoring and appropriate adjustments—such as step-up and step-down strategies—are essential for optimizing treatment outcomes.^12^ Ideally, AIT responders are expected to demonstrate continuous annual improvement throughout the three-year treatment period to achieve full efficacy. However, it remains unclear whether this consistent improvement is common in routine clinical practice. Most research to date has focused on predictors of AIT outcomes, with limited attention to clinical response trajectories over the course of 3 years. Understanding how these trajectories unfold is crucial, as it could help identify optimal intervention time points, ultimately enhancing the effectiveness of AIT.

To address this, we analyzed a large-scale data from The Real World Research Alliance for Chinese Allergic Rhinitis and Asthma Allergen Immunotherapy, a comprehensive initiative involving 53 centers nationwide. This project aims to collect detailed data on allergen immunotherapy to explore various clinical aspects. Our study sought to identify potential clinical response patterns to three-year AIT by examining changes in symptom scores from pre-treatment through the first, second, and third years of treatment. To ensure the results' generalizability and reliability, we further validated our findings using a separate cohort from our center.

## Methods

### Study design and patients

The Excellent Respiration® Digital Allergen Immunotherapy Management System (e-LinkCare Meditech Co., Ltd, Hangzhou, China), launched in 2015, is an application available on both computer and mobile devices that records follow-up clinical information for patients undergoing AIT. This system has been utilized in approximately 100 centers across China. To further enhance data collection and analysis, the Real-World Research Alliance for Chinese Allergic Rhinitis and Asthma Allergen Immunotherapy (RRAC-AIT) was established on February 10, 2022. This alliance, comprising 53 well-resourced centers nationwide, is dedicated to participating in and sharing data derived from the Excellent Respiration system. The goal is to gather large-scale real-world data and explore novel clinical aspects of AIT, ultimately contributing to better control of allergic diseases in this field.

We screened patients undergoing AIT since 2015 using raw data from the Excellent Respiration system across 53 centers participating in the RRAC-AIT initiative. To minimize the impact of product heterogeneity (eg, different brands and allergen extracts) on data interpretation, we focused on patients with house dust mite (HDM) allergy who were receiving HDM AIT administered subcutaneously with standardized depot formulations (Alutard SQ, ALK, Denmark). The inclusion criteria were: (1) a diagnosis of HDM-allergic asthma and/or allergic rhinitis;^13^^,^^14^ (2) completion of a three-year AIT regimen; and (3) availability of symptom score values recorded at 4 cross-sectional time points: baseline, 1 year, 2 years, and 3 years of AIT. HDM-allergic asthma was defined as follows: (1) A confirmed diagnosis of asthma, established by an expert physician in accordance with the *Global Initiative for Asthma (GINA)* guidelines. The diagnosis was supported by objective evidence of variable airflow limitation, defined as meeting at least 1 of the following criteria: a peak expiratory flow (PEF) variation of ≥20% over a 2-week period, a bronchodilator reversibility of ≥12% and >200 mL in FEV_1_, or airway hyperresponsiveness demonstrated by a methacholine PC_20_ ≤ 8 mg/mL (2) Objective evidence of HDM allergy, defined as fulfilling all of the following: a positive skin prick test to HDM, elevated serum HDM-specific IgE (sIgE) levels, and a consistent clinical history of symptom exacerbation triggered by HDM exposure.

We initially performed an exploratory analysis using data from 52 centers (Alliance cohort), excluding our own, to identify potential clinical response patterns that are broadly applicable across a diverse population. Following this, we validated these findings using a smaller, more detailed cohort from our own center (Guangzhou cohort) to ensure that the identified trends are both generalizable and reliable. For our center, we also required that included patients had complete clinical data, including demographic characteristics, lung function parameters, allergen indices, and airway inflammation indicators, for a more comprehensive analysis.

All centers conducted AIT in accordance with the manufacturer's instructions,^15^^,^^16^ using 4 different vials (Nos. 1–4) of standardized allergen extracts, with allergen concentrations increasing tenfold from 100 to 10,000 SQ-U/mL. The up-dosing phase involved weekly injections, with volumes of 0.2, 0.4, and 0.8 mL for vials 1–3, and 0.1, 0.2, 0.4, 0.8, and 1.0 mL for vial 4, ultimately reaching a maintenance dose of 100,000 SQ-U.

Before beginning AIT in daily clinical practice, all patients were thoroughly informed about the AIT procedure, the follow-up plan using the Excellent Respiration system, and were required to provide written informed consent. This consent included acknowledgment of the procedures and potential treatment adverse reactions, including local or systemic allergic reactions, as well as authorization for the use of their clinical data for research purposes. To ensure patient confidentiality, all data used in this study were anonymized. The study was approved by the Ethics Review Board [IRB: 2022–76] at the First Affiliated Hospital of Guangzhou Medical University, the leading center.

### Assessment of AIT effectiveness

Patients with asthma frequently exhibit comorbid conditions such as allergic rhinitis, conjunctivitis, and dermatitis.^17^, ^18^, ^19^ AIT may influence these concurrent allergic conditions. To evaluate the overall effectiveness of AIT comprehensively, we assess a broad range of 14 allergic symptoms. These symptoms include 4 related to asthma (shortness of breath, wheezing, cough, and chest tightness), 5 associated with allergic rhinitis (sneezing, nasal itching, nasal congestion, runny nose, and postnasal drip), 3 symptoms of conjunctivitis (watery eyes, itching of the eyes, and eye swelling), and 2 symptoms of dermatitis (urticaria and pruritus). The severity of these symptoms is quantified using the Visual Analog Scale (VAS),^20^ which ranges from 0 (indicating no symptoms) to 10 (indicating the most severe symptoms). VAS assessments are conducted at 4 cross-sectional time points: prior to the initiation of AIT, and at 1 year, 2 years, and 3 years of treatment. The total VAS values at these intervals serve as primary measures of AIT effectiveness, including the total VAS values at the initiation of AIT (tVAS_0w_), and at 1 year (tVAS_1y_), 2 years (tVAS_2y_), and 3 years (tVAS_3y_) of treatment.

Medication scores were only available in the Guangzhou cohort. These scores were recorded based on the GINA treatment steps^14^(with scores ranging from 1 to 5 corresponding to steps 1 through 5), along with the use of nasal corticosteroids, topical corticosteroids, and antihistamines (each scored as 1 if used, 0 if not). A responder to AIT in the Guangzhou cohort was defined as an individual who demonstrated at least a 30% reduction in the combined symptom and medication score (ΔcSMS) from baseline to 3 years of AIT. Recognizing that symptom scores and medication scores do not carry equal weight, ΔcSMS was calculated as the average of the change rates in symptom score (ΔSS) and medication score (ΔMS). The formula for ΔcSMS is as follows:

$$\Delta \text{SMS}=\frac{\Delta \text{SS}+\Delta \text{MS}}{2}$$

### Cluster analysis

This study aimed to identify distinct clinical response patterns to 3y-AIT in routine daily practice. We incorporated 2 types of values into the cluster analysis: (1) D-values: These represent the absolute changes in mean VAS scores across specific intervals: from the initiation of AIT to 1 year (D1), from 1 year to 2 years (D2), and from 2 years to 3 years (D3). D-values indicate the extent of symptom improvement or deterioration; (2) Change Percentages: These represent the relative changes in mean VAS scores over the same intervals (D1, D2, and D3). Change percentages reflect the degree of improvement or worsening between the intervals.

For class discovery and clustering validation, we utilized consensus clustering, a resampling-based method.^21^ Euclidean distance was employed to determine the similarity between participants. Clustering was performed using the k-means method. To assess cluster stability, bootstrapping was conducted by randomly removing 20% of the data and repeating the clustering process 1000 times. Cluster stability was evaluated by examining the cumulative distribution function (CDF), which was considered stable when the middle portion of the CDF was flat. To further confirm the optimal number of clusters, we employed 26 criteria using the R package NbClust, which aids in determining the number of clusters and provides the best clustering scheme.^22^

### Statistical analysis

Statistical analysis was performed utilizing the SPSS software package (version 22.0; IBM Corp, Armonk, NY). Continuous variables are presented as numbers (%), median (interquartile range), or mean (standard deviation). Normality testing was employed to determine whether the data adhered to a normal distribution. Comparisons of continuous endpoints between groups were calculated based on the variable normality assumptions using independent-sample t-tests or Mann-Whitney U tests, or ANOVA or Kruskal-Wallis tests. Categorical endpoints were analyzed using a χ2 test. Wilcoxon matched-pairs singed rank test was used to compare the effectiveness of AIT in 2 related intervals. Receiver operating characteristic (ROC) curves were utilized to assess the predictive capability of clinical parameters for identifying responders to AIT. Statistical significance was defined as a p value of <0.05.

## Results

### Patient screening and cohort selection

**Fig. 1:**
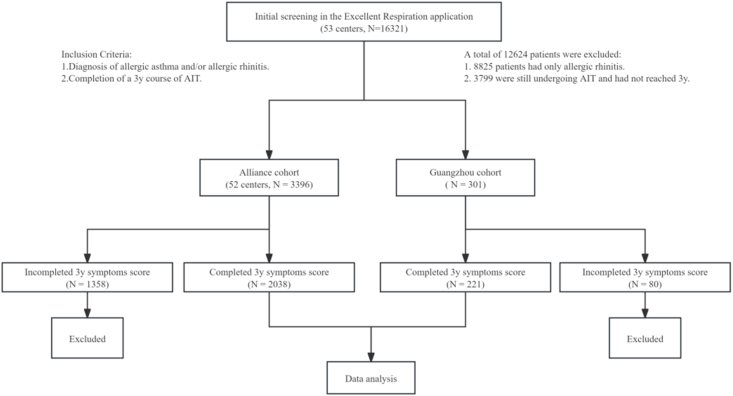
Flowchart of the study population selection and inclusion process.

A total of 16321 patients were screened using the Excellent Respiration application from 53 centers. Of these, 12624 patients were excluded: 8825 patients had only allergic rhinitis, and 3799 were still undergoing AIT and had not yet reached the 3-year duration. This left 3396 patients in the Alliance cohort and 301 patients in the Guangzhou cohort. Further exclusions were made for 1358 patients in the Alliance cohort and 80 patients in the Guangzhou cohort who had not completed the 3-year symptom score assessments. Ultimately, 2038 patients from the Alliance cohort and 221 patients from the Guangzhou cohort were included in the final analysis. The flowchart is presented in Fig. 1.

### Overall efficacy of 3y-AIT

**Fig. 2:**
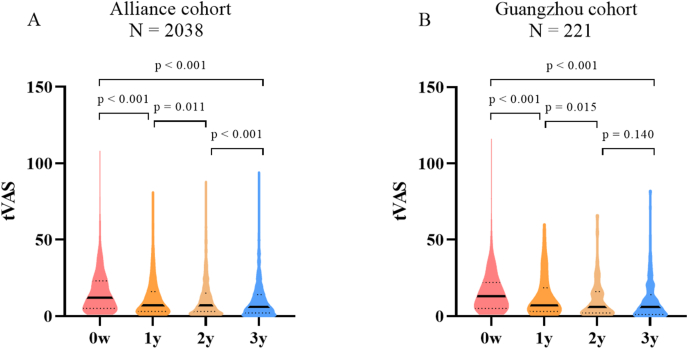
Effectiveness of 3-year allergen immunotherapy in the Alliance and Guangzhou cohorts. Abbreviations: w: week, y: year, tVAS: total Visual Analog Scale score.

Consistent with previous results from RCTs, AIT showed significant improvement in disease conditions for asthmatic patients after 3y-AIT in both the Alliance and Guangzhou cohorts. This is evidenced by the significantly lower values of tVAS_3y_ compared to tVAS_0w_ (p < 0.001 for both cohorts) (Fig. 2A and B).

### Cluster analysis for identifying clinical response trajectories

**Fig. 3:**
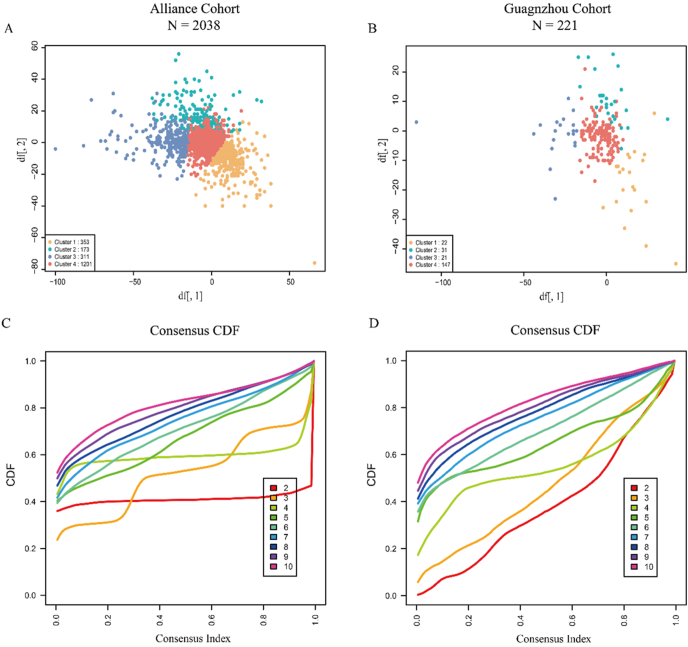
Cluster analysis of clinical response patterns in the Alliance and Guangzhou cohorts. (A) and (B) illustrate the distribution of identified clusters in the Alliance and Guangzhou cohorts, respectively. (C) and (D) display the cluster stability, as assessed by examining CDF. Stability is indicated by a flat middle portion of the CDF. Abbreviations: CDF, cumulative distribution function.

In our initial analysis of symptom score changes across different AIT periods within the Alliance cohort, we identified 4 distinct clusters of clinical response trajectories: Cluster 1 (353 patients, 17.32%), Cluster 2 (173 patients, 8.49%), Cluster 3 (311 patients, 15.26%), and Cluster 4 (1201 patients, 58.93%) (Fig. 3A). To validate these findings, we analyzed the Guangzhou cohort, where 4 similar clusters of clinical response trajectories were identified: Cluster 1 (22 patients, 9.95%), Cluster 2 (31 patients, 14.03%), Cluster 3 (21 patients, 9.50%), and Cluster 4 (147 patients, 66.52%) (Fig. 3B). The selection of 4 clusters was deemed optimal based on the evaluation of 26 clustering scheme criteria in both the Alliance and Guangzhou cohorts (Supplementary Fig. 1A–B). Cluster stability was further validated by cumulative distribution function (CDF) analysis, which showed a flat middle portion of the CDF in the Alliance cohort (Fig. 3C) and a moderately flat curve in the Guangzhou cohort (Fig. 3D).

### Clinical response trajectories in 4 clusters

**Fig. 4:**
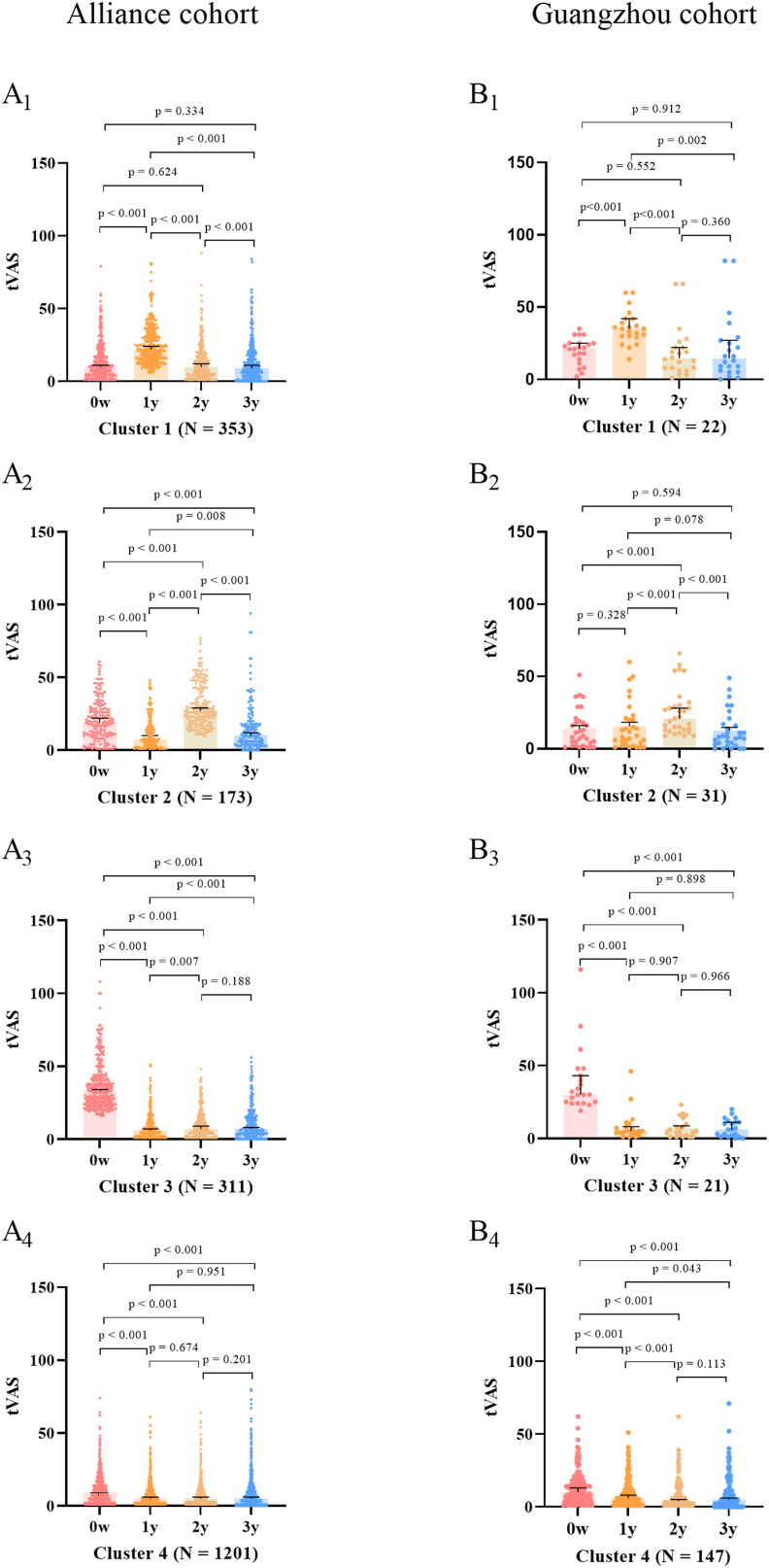
Symptom scores across different treatment periods in various clusters in the Alliance (A1–A4) and Guangzhou cohorts (B1–B4). Abbreviations: w, week; y, year; tVAS, total Visual Analog Scale score.

In Cluster 1 of the Alliance cohort, tVAS_1y_ was significantly higher than tVAS_0w_, tVAS_2y_, and tVAS_3y_ (all p < 0.001). However, tVAS_1y_ did not significantly differ from tVAS_2y_ (p = 0.624) or tVAS_3y_ (p = 0.334). This cluster reveals a clinical response pattern characterized by a worsening of symptoms in the first year, followed by improvement in the second year compared to the first, with no significant difference in symptoms by the third year compared to baseline (Fig. 4 A1).

In Cluster 2 of the Alliance cohort, although tVAS_2y_ was significantly higher than tVAS_0w_, tVAS_1y_, and tVAS_3y_ (all p < 0.001), tVAS_0w_ was still significantly higher than tVAS_3y_ (p < 0.001). This cluster is characterized by an initial improvement in symptoms during the first year, a worsening in the second year, followed by rapid improvement in the third year, ultimately leading to an overall improvement in symptoms after 3 years of AIT (Fig. 4 A2).

For Clusters 3 and 4 of the Alliance cohort, although the value of tVAS_0w_ in Cluster 3 were higher than those in Cluster 4, the tVAS_0w_ values in both clusters were significantly higher than their respective tVAS_1y_, tVAS_2y_, and tVAS_3y_ values (all p < 0.001). There were no significant differences among tVAS_1y_, tVAS_2y_, and tVAS_3y_ within each cluster. Cluster 3, with higher baseline symptom scores, and Cluster 4, with lower baseline scores, both reveal a clinical response pattern characterized by significant symptom improvement after 1 year of treatment, with sustained symptom control over the subsequent 2 years (Fig. 4 A3-4).

**Fig. 5:**
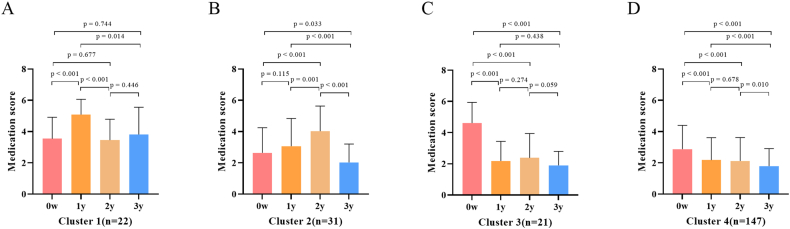
Medication scores across different treatment periods in various clusters within the Guangzhou cohort (A–D). Abbreviations: w, week; y, year;

We further validated these findings in the Guangzhou cohort, where we observed similar clinical response trajectories (Fig. 4 B1-4). Additionally, we analyzed the medication scores across the 4 clusters in the Guangzhou cohort and found similar trends aligned with the changes in symptom scores. Cluster 1 showed an increase in medication use during the first year of AIT, and the dosage did not significantly decrease after 3 years of AIT. Similarly, Cluster 2 exhibited increased medication use in the second year, with no significant reduction in dosage after 3 years of AIT. In contrast, Clusters 3 and 4 both demonstrated a decrease in medication use during the first year of AIT, with a sustained and significant reduction in medication dosage after 3 years of AIT (Fig. 5A–D).

### Clinical features of 4 clusters

In the Alliance cohort, no significant differences in demographic characteristics were observed among the clusters. However, tVAS_0w_ values were significantly different across the clusters (p < 0.001), with Cluster 3 exhibiting the highest tVAS_0w_ compared to the others (all p < 0.05) (Table 1).

**Table 1:** Characteristics of subjects in the 4 clusters of the alliance cohort.

Characteristics	Overall (N = 2038)	Cluster 1 (N = 353)	Cluster 2 (N = 173)	Cluster 3 (N = 311)	Cluster 4 (N = 1201)	p
Age (years)	12.00 (8.00, 23.75)	11.00 (8.00, 19.00)	14.00 (10.00, 23.00)	11.00 (9.00, 20.00)	13.00 (8.00, 25.00)	0.169
Children (n,%)^#^	1338.00 (65.65)	238.00 (67.42)	115.00 (66.47)	219.00 (70.42)	766.00 (63.78)	0.136
Adults (n,%)^#^	700.00 (34.35)	115.00 (32.58)	58.00 (33.53)	92.00 (29.58)	435.00 (36.22)	
Gender						
Female (n,%)	772.00 (37.88)	134.00 (37.96)	70.00 (40.46)	105.00 (33.76)	463.00 (38.55)	0.397
Male (n,%)	1266.00 (62.12)	219.00 (62.04)	103.00 (59.54)	206.00 (66.24)	738.00 (61.45)	
Body mass index∗	20.57 (17.34, 23.79)	20.83 (17.95, 23.31)	21.22 (17.80, 24.03)	20.51 (18.13, 23.19)	20.36 (16.87, 24.09)	0.371
tVAS_0w_ score∗	12.00 (5.00, 23.00)	10.00 (5.00, 20.00)^abc^	18.00 (9.00, 29.00)^de^	29.00 (20.00, 40.00)^f^	10.00 (4.00, 17.00)	<0.001

The symbols "∗" and "&", and "#" indicate data representation as median (interquartile range) and percentage (%); Comparisons among clusters were conducted using Kruskal-Wallis tests. Abbreviations:a: tVAS0w: total Visual Analog Scale score of allergic symptoms before AIT. Note: a: p < 0.05 compared between Cluster 1 and Cluster 2; b: p < 0.05 compared between Cluster 1 and Cluster 3; c: p < 0.05 compared between Cluster 1 and Cluster 4; d: p < 0.05 compared between Cluster 2 and Cluster 3; e: p < 0.05 compared between Cluster 2 and Cluster 4; f: p < 0.05 compared between Cluster 3 and Cluster 4

Similarly, in the Guangzhou cohort, tVAS_0w_ values were also significantly different across the clusters (p < 0.001), with Cluster 3 showing the highest tVAS_0w_ values. Additionally, medication scores were significantly different across the clusters (p < 0.001), with Cluster 3 again exhibiting the highest values. We also noted that the proportion of subjects exposed to tobacco from a family member was highest in Cluster 1 compared to the other clusters (p = 0.003). Furthermore, the proportion of subjects experiencing respiratory infections more than once per year during the 3y-AIT was highest in Cluster 2 (p < 0.001). However, no other pretreatment clinical parameters and demographic characteristics were found to differ significantly across the 4 clusters (Table 2).

**Table 2:** Characteristics of subjects in the 4 clusters of the Guangzhou cohort.

Characteristics	Overall (N = 221)	Cluster 1 (N = 22)	Cluster 2 (N = 31)	Cluster 3 (N = 21)	Cluster 4 (N = 147)	p
Age (years)	19.00 (10.00, 29.00)	14.50 (9.00, 28.00)	25.00 (14.00, 31.50)	21.00 (13.00, 34.00)	16.00 (10.00, 27.00)	0.133
Children (n,%)^#^	104.00 (47.06)	12.00 (54.55)	9.00 (29.03)	9.00 (42.86)	74.00 (50.34)	0.150
Adults (n,%)^#^	117.00 (52.94)	10.00 (45.45)	22.00 (70.97)	12.00 (57.14)	73.00 (49.66)	0.150
Gender						
Female (n,%)^#^	86.00 (38.91)	9.00 (40.91)	14.00 (45.16)	11.00 (53.38)	52.00 (35.37)	0.404
Male (n,%)^#^	135.00 (61.09)	13.00 (59.09)	17.00 (54.84)	10.00 (47.62)	95.00 (64.63)	0.404
Body mass index∗	20.31 (17.98, 23.15)	18.77 (17.92, 21.98)	20.81 (18.43, 23.19)	20.62 (18.38, 23.12)	20.65 (17.88, 23.19)	0.845
Tobacco exposure from family members (n,%)^#^	27.00 (12.22)	8.00 (36.36)^abc^	2.00 (6.45)	1.00 (4.76)	16.00 (10.88)	0.003
Respiratory infections >1/year (n,%)^#^	21.00 (9.50)	1.00 (4.55)	9.00 (29.03)^de^	1.00 (4.76)	10.00 (6.8)	<0.001
Acute exacerbation >1/year (n,%)^#^	26.00 (11.76)	4.00 (18.18)	3.00 (9.68)	0.00 (0.00)	19.00 (12.93)	0.262
tVAS_0w_ score∗	13.00 (5.00–22.00)	21.50 (14.75–25.00)^c^	25.00 (14.00–31.50)	30.00 (25.00–43.00)^f^	10.00 (5.00–16.00)	**<0.001**
Medication score∗	3.00 (2.00–4.00)	3.00 (2.00–4.00)^bc^	3.00 (1.00–4.50)^de^	4.00 (3.00–6.00)^f^	3.00 (2.00–4.00)	0.002
FeNO (ppb)∗	75.00 (31.00–125.00)	87.00 (73.50–147.25)	51.00 (27.00–123.00)	123.00 (46.00–186.00)	70.00 (31.00–114.50)	0.121
Total IgE (kU/l)∗	435 (169.00–855.00)	490.00 (161.50–1378.50)	410.00 (234.50–710.50)	694.00 (322.00–921.00)	417.00 (149.00–833.00)	0.428
sIgE of Der-p (kU/l)∗	36.68 (12.72–77.01)	37.18 (16.74–80.13)	38.12 (21.59–76.45)	46.44 (34.13–100.00)	31.97 (9.72–66.57)	0.124
sIgE of Der-f (kU/l)∗	38.8 (11.25–82.48)	43.09 (23.20–93.40)	37.69 (12.73–61.05)	63.25 (28.84–100.00)	36.98 (6.73–80.48)	0.185
sIgE of Der-p/Total IgE∗	0.07 (0.03–0.15)	0.06 (0.03–0.11)	0.12 (0.03–0.19)	0.08 (0.05–0.16)	0.06 (0.03–0.14)	0.334
sIgE of Der-f/Total IgE∗	0.07 (0.03–0.16)	0.08 (0.03–0.18)	0.09 (0.03–0.19)	0.10 (0.03–0.13)	0.06 (0.04–0.17)	0.969
(sIgE of Der-p + sIgE of Der-f)/Total IgE∗	0.17 (0.07–0.32)	0.17 (0.06–0.27)	0.22 (0.07–0.40)	0.18 (0.11–0.29)	0.14 (0.07–0.32)	0.576
Parameters of lungfunction						
FEV_1_ (%predicted)∗	91.90 (86.50–99.20)	89.65 (85.53–94.65)	88.20 (84.10–102.60)	89.20 (83.70–92.20)	92.00 (86.90–92.50)	0.214
FEF_25-75_ (%predicted)∗	74.60 (52.00–93.10)	82.95 (50.33–91.68)	86.20 (53.80–98.10)	80.90 (62.20–98.40)	72.30 (51.95–89.95)	0.480
FVC (%Predicted)∗	99.90 (90.0–106.80)	98.30 (88.95–104.28)	96.90 (89.30–102.60)	99.30 (85.60–102.50)	101.20 (91.15–109.20)	0.271
FEV_1_/FVC (%predicted)∗	84.30 (76.50–90.00)	84.90 (62.23–91.62)	83.10 (77.25–95.10)	87.90 (75.20–89.50)	83.60 (77.34–89.35)	0.384
Peripheral eosinophil count (10^9^/L)∗	0.61 (0.38–0.94)	0.70 (0.60–1.06)	0.61 (0.29–0.89)	0.70 (0.56–0.94)	0.58 (0.35–0.93)	0.222
Induced sputum						
Neutrophil (%)∗	40.60 (18.50–61.80)	50.95 (21.45–68.15)	38.70 (12.05–56.45)	27.00 (12.50–58.30)	43.00 (20.65–63.60)	0.187
Eosinophil (%)∗	5.00 (1.50–9.90)	6.70 (2.65–8.58)	6.80 (1.90–9.65)	1.30 (0.80–7.40)	4.90 (1.50–11.45)	0.100
Lymphocyte (%)∗	2.30 (1.40–3.70)	2.55 (1.35–3.53)	1.67 (0.85–3.50)	2.20 (1.50–4.20)	2.40 (1.40–3.70)	0.556
Macrophage (%)∗	51.00 (27.80–70.40)	36.75 (25.65–66.63)	55.50 (34.80–74.10)	64.50 (37.00–77.00)	48.10 (26.95–69.05)	0.265

The symbols "∗" and "&", and "#" indicate data representation as median (interquartile range) and percentage (%); Comparisons among clusters were conducted using Kruskal-Wallis tests. Abbreviations:a: tVAS0w: total Visual Analog Scale score of allergic symptoms before AIT; FEV_1_: forced expiratory flow in 1 s; FVC: forced vital capacity; FEF_25-75_: forced expiratory flow between 25 and 75% of vital capacity; IgE: immunoglobulin E; FeNO: Fractional exhaled nitric oxide. Note: a: p < 0.05 compared between Cluster 1 and Cluster 2; b: p < 0.05 compared between Cluster 1 and Cluster 3; c: p < 0.05 compared between Cluster 1 and Cluster 4; d: p < 0.05 compared between Cluster 2 and Cluster 3; e: p < 0.05 compared between Cluster 2 and Cluster 4; f: p < 0.05 compared between Cluster 3 and Cluster 4

### Predictors of AIT responders

**Fig. 6:**
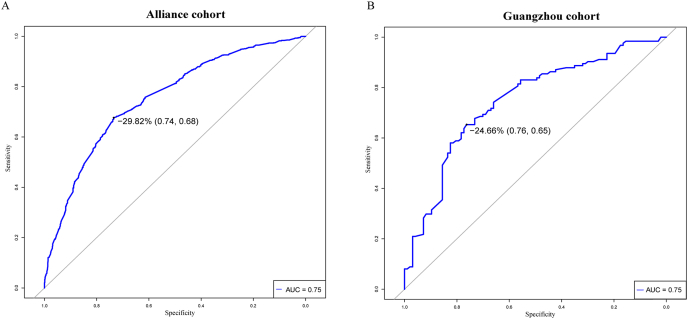
ROC analysis of predictors of AIT responders in the Alliance (plot A) and Guangzhou cohorts (plot B).

In our analysis of 4 clinical response trajectories, the effectiveness of the first year of AIT emerged as a strong predictor of the overall 3-year effectiveness. ROC analysis showed that the change in VAS from baseline to 1 year (ΔVAS_1y_) achieved an AUC of 0.75, with an cutoff of 29.40% (specificity: 74.00%, sensitivity: 68.00%) in the Alliance cohort. Similarly, in the Guangzhou cohort, ΔVAS_1y_ also showed an AUC of 0.75, with a cutoff of 24.66% (specificity: 76.00%, sensitivity: 65.00%) (Fig. 6).

### Comparison of responders and non-responders across the 4 clusters

In the Guangzhou cohort, responders were defined as those showing at least a 30% reduction in SMS. The distribution of responders and non-responders across clusters was as follows: Cluster 1 had 15 non-responders (68.18%) and 7 responders (31.82%); Cluster 2 had 16 non-responders (51.62%) and 15 responders (48.39%); Cluster 3 had 6 non-responders (28.57%) and 15 responders (71.43%); and Cluster 4 had 58 non-responders (39.46%) and 89 responders (60.54%). The portion of responders were the lowest in the Cluster 1 and Cluster 3 presented the highest responders rate among 4 clusters (p = 0.001). However, we did not observe a difference bwetween cluster 3 and 4 (p = 0.116) (eFig. 1A).

Further classification of responders into fair-responders (30% ≤ ΔSMS <60%) and super-responders (ΔSMS >60%) revealed the following: Cluster 1 had 2 fair-responders (50.00%) and 2 super-responders (50.00%); Cluster 2 had 10 fair-responders (71.43%) and 4 super-responders (28.57%); Cluster 3 had 3 fair-responders (18.75%) and 13 super-responders (81.25%); and Cluster 4 had 42 fair-responders (47.19%) and 47 super-responders (52.81%). Cluster 3 showed highest super-responder rate among 4 clusters (p = 0.037) (eFig. 1B). Among the 3 groups (non-responders, fair-responders, and super-responders), non-responders had the highest rate of tobacco exposure from family members (p = 0.001). However, no significant differences were observed in other clinical parameters across the 4 clusters (see eTable 1).

## Discussion

Beyond focusing on AIT efficacy, this retrospective study represents the first large-scale exploration of clinical response trajectories of 3y-AIT in routine practice. Cluster analysis identified 4 distinct clusters of clinical response trajectories, each with unique characteristics. Cluster 1 showed initial symptom worsening during the first year, with no response to AIT by the third year. Cluster 2 experienced symptom deterioration in the second year, followed by significant recovery in the third year, ultimately achieving a positive response after 3 years. Clusters 3 and 4, which were distinguished by higher and lower baseline symptom severity, respectively, both demonstrated marked symptom improvement after the first year and sustained control throughout the remaining 2 years. Validation in a smaller, independent cohort confirmed the generalizability and reliability of these clinical response trajectories. Recognized these trajectories and their associated clinical outcomes provides a “dynamic understanding” of the 3-year AIT process and how final outcomes are achieved.

Clinical response is expected to be the most direct indicator of AIT effectiveness, influenced by factors including allergen extract-immune response interactions, medication adherence, treatment intensity, environmental control, and unexpected events like upper and lower airway infections.^10^^,^^23^^,^^24^ Given these complexities, heterogeneous response trajectories to 3y-AIT at the individual level are anticipated. Despite this expected heterogeneity, we identified 4 primary clinical response trajectories with distinct characteristics. Cluster 1 was unique in showing no benefit from AIT, with symptoms worsening after 1 year of treatment. In contrast, Clusters 2, 3, and 4 demonstrated positive responses to AIT after 3 years, though with differing trajectories. Cluster 2 experienced a worsening of symptoms during the second year, Cluster 3 started with more severe symptoms, and Cluster 4 began with milder symptoms. This retrospective analysis reveals real-world clinical response patterns under in daily practice. Both Cluster 1 and Cluster 2 experienced symptom worsening, while Clusters 3 and 4 can be considered examples of successful AIT management. These findings highlight 2 key points: (1) the need to identify better strategies to prevent symptom worsening, as seen in Cluster 2; and (2) the evidence provided by Cluster 1 suggests that if symptoms worsen after 1 year of AIT, discontinuation of treatment may be warranted, as the long-term efficacy is often suboptimal.

In this study, although we identified 4 primary clinical response trajectories to 3-year AIT, predicting these trajectories based on pre-treatment parameters remains challenging. No significant differences were observed among the 4 clusters in terms of allergic parameters, airway eosinophilic levels, or lung function indices. This result was somewhat expected, as no reliable clinical indicators have yet been established to predict AIT efficacy, and these routine clinical parameters appear to have limited predictive value for AIT response trajectories. However, we did find potential clues that may contribute to the differentiation of these clusters. For instance, subjects in Cluster 1 were more likely to be exposed to tobacco smoke from family members, while those in Cluster 2 had a higher incidence of airway infections. Environmental pollution is widely recognized for its detrimental effects on asthma development and progression.^25^^,^^26^ However, the importance of incorporating personal and environmental measures, such as avoiding smoking, into long-term AIT management has not been fully emphasized.^7^ Beyond considering an individual's allergic history and immune response to allergen stimulation, environmental factors may also contribute to a decline in AIT efficacy. Compared with personal smoking habits, occupational allergen exposure, or outdoor air pollution, exposure to secondhand tobacco smoke at home is a frequently overlooked environmental factor. Based on our findings, we have stronger evidence to highlight that avoiding both active smoking and secondhand smoke exposure should be regarded as an integral component of AIT management, as these factors may substantially influence therapeutic efficacy and long-term treatment outcomes. Additionally, airway infections are well-known for exacerbating asthma,^27^^,^^28^ as observed in Cluster 2, where frequent infections were associated with a less stable response to 3-year AIT. Interestingly, our previous studies observed that patients receiving AIT exhibited better clinical outcomes after COVID-19 infection compared with non-AIT patients, and our mechanistic investigations further demonstrated enhanced CD8^+^ T-cell activity following AIT. These findings suggest that AIT may exert dual benefits by modulating both allergen-specific immune tolerance and antiviral immune responses. Therefore, in patients who continue to experience recurrent infections during AIT, such infections might indicate an insufficient immunomodulatory shift from Th2-dominant toward Th1-balanced immunity, which could potentially serve as a predictor of suboptimal AIT response.

Unlike RCTs, which impose strict inclusion and exclusion criteria requiring pretreatment parameters to remain relatively uniform,^29^^,^^30^ daily clinical practice encounters patients with a wide range of clinical characteristics. One of the most heterogeneous traits is their symptom severity. It is reasonable to assume that more severe allergic symptoms in allergen-driven asthma are associated with a higher level of allergen sensitization, which might lead to a greater likelihood of benefiting from AIT.^31^ Currently, there is no data addressing whether patients with more severe symptoms experience greater benefit from AIT compared to those with less severe symptoms. In this study, clusters 3 and 4 demonstrated effective AIT trajectories, indicating that both high and relatively low baseline symptom severities showed significant improvement over 3-AIT, with no difference in the rate of responders between these 2 clusters. This observation carries important clinical implications. In daily practice, some patients present with mild symptoms that are well-controlled under regular medication but remain unable to reduce pharmacotherapy due to continuous allergen exposure. For these patients, AIT is often initiated to decrease medication dependence and enhance long-term disease stability.Our findings suggest that, provided patients meet the established indications for AIT, the therapy remains worthwhile even for those with good baseline control, as it may further optimize immune regulation and prevent future disease exacerbations. This evidence helps to mitigate previous concerns that patients with well-controlled symptoms may show limited improvement when evaluated solely using symptom-based scores.

The heterogeneous responses to 3-year AIT reflect not only different treatment response trajectories but also varying levels of allergen desensitization, which may be directly correlated with the long-term development of allergen tolerance.^8^ Currently, the assessment of allergen desensitization primarily relies on objective methods,^4^ such as the VAS score, which can be significantly influenced by pretreatment disease severity. Indeed, we observed that super-responders to AIT had higher symptom and medication scores before starting AIT compared to standard responders. In our study, we were unable to identify other pretreatment clinical parameters that could differentiate super-responders from standard-responders. However, we believe that super-responders to AIT represent an exceptionally favorable response to the therapy. Investigating this specific group of patients, rather than the general population of AIT responders, could provide valuable insights into the mechanisms and clinical management of AIT. Although our results delineate the real-world heterogeneity of AIT response trajectories, identifying reliable predictors of these trajectories remains a major challenge. In our previous work, we demonstrated that the first-year AIT response could sensitively predict the therapeutic outcome at year 3.^32^ This finding was further validated in the present large-scale cohort, confirming that such predictive value remains robust even in the context of pronounced response heterogeneity. Therefore, early treatment response to AIT could serve as a practical “pilot test” for the full three-year regimen: a favorable early response suggests effective allergen desensitization, adequate disease management, and beneficial environmental interventions, collectively supporting sustained efficacy throughout the course of therapy.

This large-scale, multicenter study identifies distinct response trajectories observed in routine clinical practice. The findings were first discovered in an exploratory dataset and subsequently validated in an independent cohort, which enhances the robustness of our conclusions. However, several limitations should be acknowledged. Due to the retrospective nature of this real-world, symptom-based registry, the availability of detailed longitudinal clinical data—such as lung function parameters and markers of airway eosinophilic inflammation—was limited. Similarly, biological information, including allergen-specific IgG4 levels, immune cell profiles, and cytokine or inflammatory mediator data, was not available. While we were able to identify 4 main treatment response trajectories over 3 years of AIT, we could not determine the specific factors driving these patterns or the underlying immune mechanisms. Further studies incorporating comprehensive clinical and biomarker assessments are warranted to identify variables that may predict or contribute to favorable long-term AIT responses. In addition, although our initial database included a large number of patients receiving HDM subcutaneous immunotherapy, only about 15% met the strict inclusion criteria for this analysis, which may have introduced selection bias. Despite internal validation within our local cohort, external validation of these response trajectories in other populations and geographical regions remains necessary to confirm their generalizability. It is also noteworthy that no conventional clinical parameters clearly distinguished among the 4 trajectories. This finding is consistent with the general understanding that routine clinical indicators have limited predictive value for AIT outcomes. Finally, although this study identified distinct patterns of treatment response, the retrospective design does not allow us to propose intervention strategies to modify or redirect these trajectories. Future prospective studies integrating mechanistic and interventional analyses are needed to address these important questions.

In conclusion, 4 primary treatment response trajectories with unique features were identified in 3-year AIT in a multicenter, real-world clinical setting. These findings support the overall efficacy of AIT while highlighting that disease deterioration during the first year of treatment can negatively impact the final effectiveness of 3-year AIT. Enhanced and detailed monitoring, along with timely adjustments in treatment strategies, are necessary to prevent disease deterioration and achieve optimal outcomes with 3y-AIT.

## Data availability

The datasets generated and analyzed during this study are not publicly available due to confidentiality agreements and privacy concerns regarding the participants.

## Authors' contributions

JL conceived and designed the study. All authors contributed to data acquisition. ZP and RQ handled data curation and processing. ZP, RQ, and JL conducted the data analysis. All authors participated in the interpretation of the results. ZP drafted the initial manuscript, and all authors made critical revisions throughout the development of this manuscript.

## Ethics

This study was performed in line with the principles of the Declaration of Helsinki. The study was approved by the Ethics Review Board [IRB: 2022–76] at the First Affiliated Hospital of Guangzhou Medical University, the leading center.

## Consent and permissions

All participants were informed of the research objectives, methods and objectives. All participants provided written informed consent.

## Declaration of Generative AI and AI-assisted technologies in the writing process

Nothing to disclose.

## Funding

This work was supported by the Noncommunicable Chronic Diseases-National Science and Technology Major Project (2024ZD0529900), National Natural Science Foundation of China (82161138020).

## Declaration of competing interest

The authors declare no conflict of interest in relation to this work.

## Figures and Tables

**figs1:**