# Supplementary material for: Distinct treatment response trajectories to allergen immunotherapy in allergic asthma and rhinitis: Insights from a multicenter study in routine clinical practice
Source: World Allergy Organ J. 2026 Jan 5;19(1):101168. doi: 10.1016/j.waojou.2025.101168 (PMC12811459; doi:10.1016/j.waojou.2025.101168)
Supplement: Multimedia component 1 [file mmc1.docx]

| eTable 1. Characteristics of Subjects in Different Responder Groups. | | | | |
| --- | --- | --- | --- | --- |
| Characteristics | Non- responders  (N=98) | Fair-responders  (N=57) | Super-responders  (N=66) | p |
| Age (years) | 20.00(10.00-30.00) | 15.00(8.00-27.00) | 19.00(11.50-29.75) | 0.208 |
| Children (n, %)^#^ | 43.00(43.88) | 33.00(57.89) | 28.00(42.42) | 0.161 |
| Adults (n, %)^#^ | 55.00(56.12) | 24.00(42.11) | 38.00(57.58) | 0.161 |
| Gender |  |  |  |  |
| Female (n, %)^#^ | 43(43.88) | 20.00(35.09) | 23.00(34.85) | 0.401 |
| Male (n, %)^#^ | 55(56.12) | 37.00(64.91) | 43.00(65.15) | 0.401 |
| Body mass index* | 20.20(17.99-23.48) | 20. 81(17.84-22.89) | 20.73(18.36-23.09) | 0.988 |
| Tobacco exposure from family members (n, %) ^#^ | 21.00(21.43) | 4.00(7.02) | 2.00(3.06) | 0.001 |
| Respiratory infections > 1/year (n, %)^#^ | 12.00(12.24) | 7.00(12.28) | 2.00(3.03) | 0.101 |
| Acute exacerbation > 1/year (n, %)^#^ | 17.00(17.35) | 5.00(8.77) | 4.00(6.06) | 0.064 |
| tVAS0w score* | 12.00(5.00-21.00) | 11.00(5.00-23.00) | 14.00(7.00-24.00) | 0.173 |
| Medication score* | 2.00(1.00-4.00) | 3.00(1.00-4.00) | 4.00(3.00-5.00) | <0.001 |
| FeNO (ppb)* | 80.00(31.50-128.00) | 59.00(28.00-120.00) | 72.00(36.00-120.50) | 0.566 |
| Total IgE (kU/l)* | 468.00(169.00-840.50) | 357.00(169.00-831.00) | 401.00(176.25-919.25) | 0.859 |
| sIgE of Der-p (kU/l)* | 34.60(15.04-80.85) | 38.93(12.72-81.64) | 32.28(6.79-62.77) | 0.523 |
| sIgE of Der-f (kU/l)* | 34.17(12.85-74.66) | 42.26(11.06-76.40) | 45.44(6.42-87.81) | 0.981 |
| sIgE of Der-p/Total IgE* | 0.07(0.03-0.14) | 0.09(0.04-0.17) | 0.05(0.03-0.12) | 0.168 |
| sIgE of Der-f/Total IgE* | 0.07(0.04-0.16) | 0.07(0.04-0.18) | 0.07(0.03-0.15) | 0.824 |
| (sIgE of Der-p+sIgE of Der-f)/Total IgE* | 0.17(0.07-0.31) | 0.21(0.08-0.36) | 0.13(0.07-0.28) | 0.326 |
| Parameters of lungfunction |  |  |  |  |
| FEV_1_ (%predicted)* | 91.85(85.10-99.65) | 93.60(87.90-99.40) | 89.85(85.90-98.45) | 0.411 |
| FEF_25-75_ (%predicted)* | 73.40(49.78-93.98) | 73.40(52.60-90.80) | 76.90(56.10-91.65) | 0.673 |
| FVC (%predicted)* | 99.90(90.15-106.00) | 101.60(89.70-108.50) | 98.25(89.90-106.40) | 0.839 |
| FEV_1_/FVC (%predicted)* | 83.45(75.23-88.95) | 87.40(78.80-91.30) | 83.15(76.05-89.73) | 0.198 |
| Peripheral eosinophil count (10^9^/L)* | 0.67(0.41-0.99) | 0.51(0.25-0.86) | 0.60(0.39-0.88) | 0.463 |
| Induced sputum |  |  |  |  |
| Neutrophil (%)* | 40.75(18.70-65.10) | 43.00(24.10-59.50) | 35.65(15.70-61.45) | 0.679 |
| Eosinophil (%)* | 5.50 (1.53-10.65) | 3.00(1.40-8.00) | 6.30(1.43-11.70) | 0.188 |
| Lymphocyte (%)* | 2.40(1.43-3.98) | 2.30(1.20-3.60) | 2.20(1.50-3.20) | 0.573 |
| Macrophage (%)* | 52.65(27.20-70.38) | 43.90(32.50-70.00) | 50.05(28.40-73.15) | 0.982 |

The symbols "*" and "&", and "#" indicate data representation as median (interquartile range) and percentage (%); Comparisons among clusters were conducted using Kruskal-Wallis tests. Abbreviations:a: tVAS0w: total Visual Analog Scale score of allergic symptoms before AIT; FEV_1_: forced expiratory flow in 1 second; FVC: forced vital capacity; FEF_25-75_: forced expiratory flow between 25 and 75% of vital capacity; IgE: immunoglobulin E; FeNO: Fractional exhaled nitric oxide.
